# Genetic evidence for a western Chinese origin of broomcorn millet (*Panicum miliaceum*)

**DOI:** 10.1177/0959683618798116

**Published:** 2018-09-14

**Authors:** Harriet V Hunt, Anna Rudzinski, Hongen Jiang, Ruiyun Wang, Mark G Thomas, Martin K Jones

**Affiliations:** 1 McDonald Institute for Archaeological Research, University of Cambridge, UK; 2 Research Department of Genetics, Evolution and Environment, University College London, UK; 3 Department of Archaeology and Anthropology, University of Chinese Academy of Sciences, China; 4 College of Agriculture, Shanxi Agricultural University, China; 5 Institute of Crop Germplasm Resources of Shanxi Academy of Agricultural Sciences, Key Laboratory of Crop Gene Resources and Germplasm Enhancement on Loess Plateau, Ministry of Agriculture, Shanxi Key Laboratory of Genetic Resources and Genetic Improvement of Minor Crops, China; 6 UCL Genetics Institute, University College London, UK; 7 Department of Archaeology and Anthropology, University of Cambridge, UK

**Keywords:** agricultural origins, broomcorn millet, China, domestication, early Holocene, Loess Plateau, *Panicum*, semi-arid

## Abstract

Broomcorn millet (*Panicum miliaceum*) is a key domesticated cereal that has been associated with the north China centre of agricultural origins. Early archaeobotanical evidence for this crop has generated two major debates. First, its contested presence in pre-7000 cal. BP sites in eastern Europe has admitted the possibility of a western origin. Second, its occurrence in the 7th and 8th millennia cal. BP in diverse regions of northern China is consistent with several possible origin foci, associated with different Neolithic cultures. We used microsatellite and *granule-bound starch synthase I* (*GBSSI*) genotype data from 341 landrace samples across Eurasia, including 195 newly genotyped samples from China, to address these questions. A spatially explicit discriminative modelling approach favours an eastern Eurasian origin for the expansion of broomcorn millet. This is consistent with recent archaeobotanical and chronological re-evaluations, and stable isotopic data. The same approach, together with the distribution of *GBSSI* alleles, is also suggestive that the origin of broomcorn millet expansion was in western China. This second unexpected finding stimulates new questions regarding the ecology of wild millet and vegetation dynamics in China prior to the mid-Holocene domestication of millet. The chronological relationship between population expansion and domestication is unclear, but our analyses are consistent with the western Loess Plateau being at least one region of primary domestication of broomcorn millet. Patterns of genetic variation indicate that this region was the source of populations to the west in Eurasia, which broomcorn probably reached via the Inner Asia Mountain Corridor from the 3rd millennium BC. A secondary westward expansion along the steppe may have taken place from the 2nd millennium BC.

## Introduction

Broomcorn millet (*Panicum miliaceum* L.) is significant in the history of plant domestication as a pioneering cereal, both chronologically and ecologically. It was among the world’s earliest domesticated cereals, of comparable antiquity to wheat and rice (Lu et al., 2009), and has the shortest life cycle and highest water use efficiency of any cereal (Baltensperger, 2002), enabling both its early cultivation in a wide range of ecological zones and its integration into the economy of semi-mobile agro-pastoral societies (Spengler et al., 2014). As a consequence, broomcorn millet domestication is an important proxy in addressing a range of archaeological questions regarding early agricultural societies, including the nature of the transition to agriculture in northern China – one of the world’s independent centres of agricultural innovation – and the nature and chronology of contact between early agricultural societies in Eastern and Western Eurasia (Jones et al., 2011). Specifically, the identification of broomcorn millet in the archaeobotanical record has provoked two debates. First, its reported presence prior to 7000 cal. BP at a number of sites in both northern China and eastern Europe invited explanation; whether these finds represent separate domestications, or the earliest reported contact and innovation exchange between east and west Eurasia, or neither, is yet to be resolved (Hunt et al., 2008, 2011; Jones, 2004; Motuzaite-Matuzeviciute et al., 2013). Second, the widely held view of a Yellow River origin for northern Chinese agriculture, derived from early Chinese archaeobotanical work in Cishan-Peiligang culture sites in the 1970s and 1980s, has been more recently challenged by evidence of broomcorn millet predating 6000 cal. BP from several regions and Neolithic cultures in northern China, including the western Loess Plateau (Dadiwan in Gansu), northeastern China (Xinglonggou in Inner Mongolia and Xinle in Liaoning) and the lower Yellow River valley (Yuezhuang in Shandong) (Barton et al., 2009; Ren et al., 2016; Zhao, 2011). New archaeobotanical data, combined with radiocarbon dating and broader archaeological considerations, have led to the suggestion that any or all of these regions and their associated Neolithic cultures may have been nuclei of the development of agriculture in its north Chinese centre (Cohen, 2011; Liu et al., 2009; Zhao, 2011). Their relative contribution and the complexities of their interaction remain an open question (Cohen, 2011). Such a ‘federal origin’ for farming has recently been proposed for southwest Asia (Broushaki et al., 2016), an assessment which has been supported by both the genetic and particularly the macrobotanical studies (Civáň et al., 2013; Willcox, 2013).

Here, we present new genetic data on broomcorn millet, incorporating an additional 195 landraces sampled from China. These together with previously genotyped samples provide a geographically comprehensive picture of genetic diversity across the Eurasian range of this domesticate. Using a novel statistical approach, we are able to directly address questions of both a dual eastern and western origin, and the relative contributions of north China’s subregions to the development of millet agriculture. Our genetic results are timely in the light of recent work on millet through archaeobotanical and isotopic studies in China, eastern Europe, central Asia and the Caucasus, driving the emergence of a multidisciplinary narrative of the role of this crop in Eurasian prehistory.

## Methods

### Samples

In total, 195 landrace accessions of broomcorn millet were obtained from the Chinese National Genebank (Institute of Crop Germplasm Resources, Chinese Academy of Agricultural Sciences). Accessions were chosen to provide representative geographical coverage across the provinces of China from the total accessions in the National Genebank. Details of the 195 accessions used are given as Supplementary Information Table S1, available online. Genomic DNA was extracted from 100 mg leaf tissue of a single young seedling of each accession using a Plant Genomic DNA Extraction Kit (Tiandz, Inc., Beijing, China) and quantified using 1 µl of DNA sample with an e-spect (ES-2) Micro UV-Vis Fluorescence Spectrophotometer (Malcom, Tokyo, Japan).

### Genotyping and datasets

Samples were genotyped for 16 microsatellite loci as described previously (Hunt et al., 2011, 2013). Samples from our previous dataset (Hunt et al., 2013) were included on each genotyping plate to ensure allele scoring was consistent with our previous results. The new samples were analysed as a stand-alone dataset (‘Chinese samples’, *n* = 195) and as an amalgamated dataset (‘panEurasian samples’, *n* = 341), including 146 of the samples published previously (Hunt et al., 2013). This represents all samples from our previous study except those 32 from China with minimally specific geographic location data, which were excluded.

The new samples were additionally genotyped for the three variable sites across two duplicated loci of the *granule-bound starch synthase I* (*GBSSI*) gene that control the synthesis of amylose in broomcorn millet endosperm starch. Wild-type plants have around 30% amylose in endosperm starch; waxy plants have mutations at specific combinations of the three functionally variable sites, lack endosperm amylose and have a characteristically glutinous or sticky texture on cooking (Hunt et al., 2010, 2013). DNA samples genotyped for microsatellite loci were also genotyped for the *GBSSI-S* and *GBSSI-L* loci as described previously (Hunt et al., 2013). The full genotyping dataset is available as Supplementary Information (Table S1, available online).

### Population genetic analyses

Principal components analysis was performed separately for the Chinese and panEurasian datasets using the R (R Development Team, 2016) packages *ade4* (Dray and Dufour, 2007) and *adegenet* (Jombart, 2008). Genetic clusters were modelled using a Bayesian clustering algorithm implemented in Instruct (Gao et al., 2007). Bayesian clustering uses an iterative process to identify genetic populations from the variation data for a sample set, where different numbers of populations (*K*) are modelled in independent runs of the algorithm. Simultaneously, the relative contribution of each of the *K* ancestral populations to the genetic makeup of each sampled individual is estimated. The most realistic value of *K* for the dataset is then inferred statistically (Porras-Hurtado et al., 2013). Instruct is an alternative to the widely used STRUCTURE software for Bayesian genetic clustering (Pritchard et al., 2000), but, unlike the latter, does not seek to maximise Hardy–Weinberg equilibrium, which assumes random mating. It is therefore more appropriate for a species such as *P. miliaceum*, which is strongly selfing. Ten replicate runs were performed each number of clusters (*K*) from *K* = 1 to *K* = 12, with 200,000 burn-in and 1,000,000 Markov chain Monte Carlo reps. We used CorrSieve ver. 1-6.5 (Campana et al., 2011) to determine the optimum *K*, according to the Δ*K* statistic (Evanno et al., 2005) and correlation of *Q*-matrices among multiple runs. We also checked the Deviance Information Criterion (DIC) reported by Instruct.

### Geographic origins of population expansions

**Figure 1.**
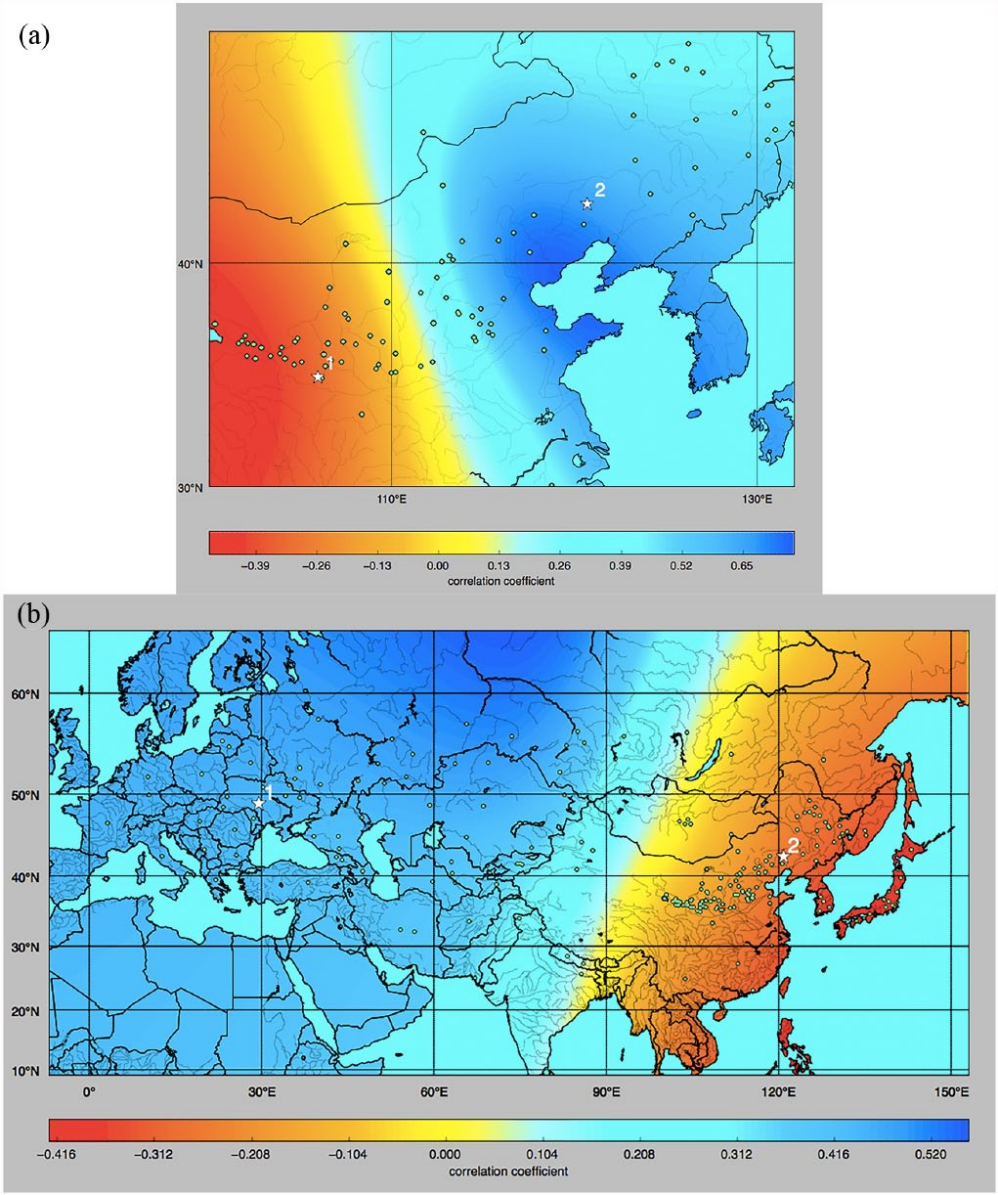
(a) Interpolated surface of correlation coefficient values between genetic diversity (unbiased heterozygosity of Chinese broomcorn millet microsatellite data recorded in kernels) and geographic distance. Red colour shows negative correlation values, gradually turning blue the more positive the correlation values become. Since genetic diversity is expected to decrease with geographic distance from the origin of an expansion, regions yielding more negative correlation values represent more plausible locations for the source of spread of broomcorn millet. Green dots show the sample locations. White stars indicate the locations of Dadiwan (1) and Xinglonggou (2). (b) Interpolated surface of correlation coefficient values between genetic diversity (unbiased heterozygosity of panEurasian broomcorn millet microsatellite data recorded in kernels) and geographic distance. Red colour shows negative correlation values, gradually turning blue the more positive the correlation values become. Since genetic diversity is expected to decrease with geographic distance from the origin of an expansion, regions yielding more negative correlation values represent more plausible locations for the source of spread of broomcorn millet. Green dots show the sample locations. White stars indicate the locations of Sokol’tsy (1) and Xinglonggou (2).

We implement a spatially explicit discriminative modelling approach to infer the geographic source location for the expansion of broomcorn millet. This model assumes a monotonic decline in genetic diversity with distance from origin location (Manica et al., 2007; Ramachandran et al., 2005). Such a decline is expected under any radial expansion process that does not involve admixture with populations already present in the regions expanded into, as genetic variation is sequentially sampled on the wavefront of the expanding population (Austerlitz et al., 1997; Klopfstein et al., 2005; Nei et al., 1975). A spatial grid of latitude and longitude ranges covering the geographic space between Europe and Japan (7°W to 153°E, 9°N to 67°N) for the panEurasian microsatellite dataset, and ranges covering China (95°E to 135°E, 30°N to 50°N) for the Chinese dataset, were searched at resolutions of 0.1 by 0.1 and 0.01 by 0.01 degrees, respectively. At each point in these searches where five or more genetic samples were present within a radius of 500 km (accepted kernels), the mean (across loci) unbiased heterozygosity was calculated (Nei, 1978). For the panEurasian dataset, 333 samples were included, and for the China-specific dataset, 188 samples were included. The grids were then re-explored with each latitude/longitude location treated as a potential origin location of broomcorn millet expansion. At each location, we recorded the Pearson’s correlation coefficient between geographic distance to the accepted kernels and local diversity at those kernels. This provided a grid of correlation values, which was then interpolated and visualised on a map. Since genetic diversity is expected to decrease with geographic distance from the origin of an expansion, regions yielding more negative correlation values represent more plausible locations for the source of spread of broomcorn millet (in red in Figure 1a and b).

**Figure 2.**
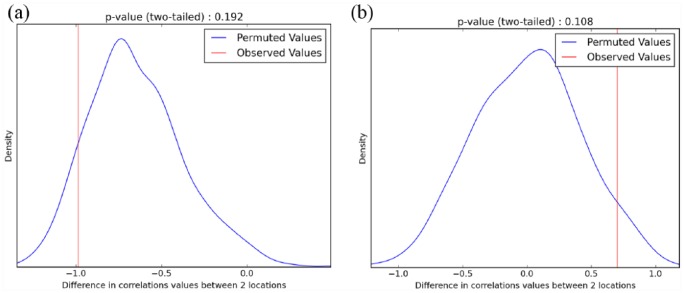
(a) Comparison of the observed difference in Pearson’s correlation coefficients (red line) between Dadiwan (‘1’ in Figure 1a) and Xinglonggou (‘2’ in Figure 1a) generated with Chinese dataset, to the distribution of those generated by permuting (randomly distributing) the site data among sample sites 1000 times (blue line). The *p* values represent the probability of obtaining the observed difference in correlation values under the null hypothesis of no geographic structure in the genetic data. This can be interpreted as a measure of how well the data favour one site over the other as a location for the source of spread of broomcorn millet, given the assumption that genetic diversity decreases with geographic distance from the origin of expansion. (b) Comparison of the observed difference in Pearson’s correlation coefficients (red line) between Sokol’tsy (‘1’ in Figure 1b) and Xinglonggou (‘2’ in Figure 1b) generated with panEurasian dataset, to the distribution of those generated by permuting (randomly distributing) the site data among sample sites 1000 times (blue line). The *p* values represent the probability of obtaining the observed difference in correlation values under the null hypothesis of no geographic structure in the genetic data. This can be interpreted as a measure of how well the data favour one site over the other as a location for the source of spread of broomcorn millet, given the assumption that genetic diversity decreases with geographic distance from the origin of expansion.

For each of the two datasets (panEurasian and China-specific), we compared two hypothesised locations of origin based on archaeobotanical evidence for early broomcorn millet; for the panEurasian dataset, we compared a Ukrainian site (Sokol’tsy) against Xinglonggou in China, and for the Chinese dataset, we tested Dadiwan in Gansu province against Xinglonggou (white stars in Figure 1a and b). To quantify support for one location to be the origin of population expansion over the other, we first calculated the difference in correlation values for the two hypothesised origin sites considered. To test if these differences were greater than expected by chance, we permuted (randomly distributed) the site data among sample sites 1000 times, and for each of these 1000 permuted datasets, we repeated the above analysis and recorded the difference in correlation values for the two hypothesised origin locations. This gives an expected distribution of difference in correlation values between each pair of sites under the null hypothesis of no geographic structure in the genetic data. Finally, we compared the differences in correlation values for the observed data with those generated from permuted data to calculate two-tailed *p* values (Figure 2a and b).

## Results

### PCA results

**Figure 3.**
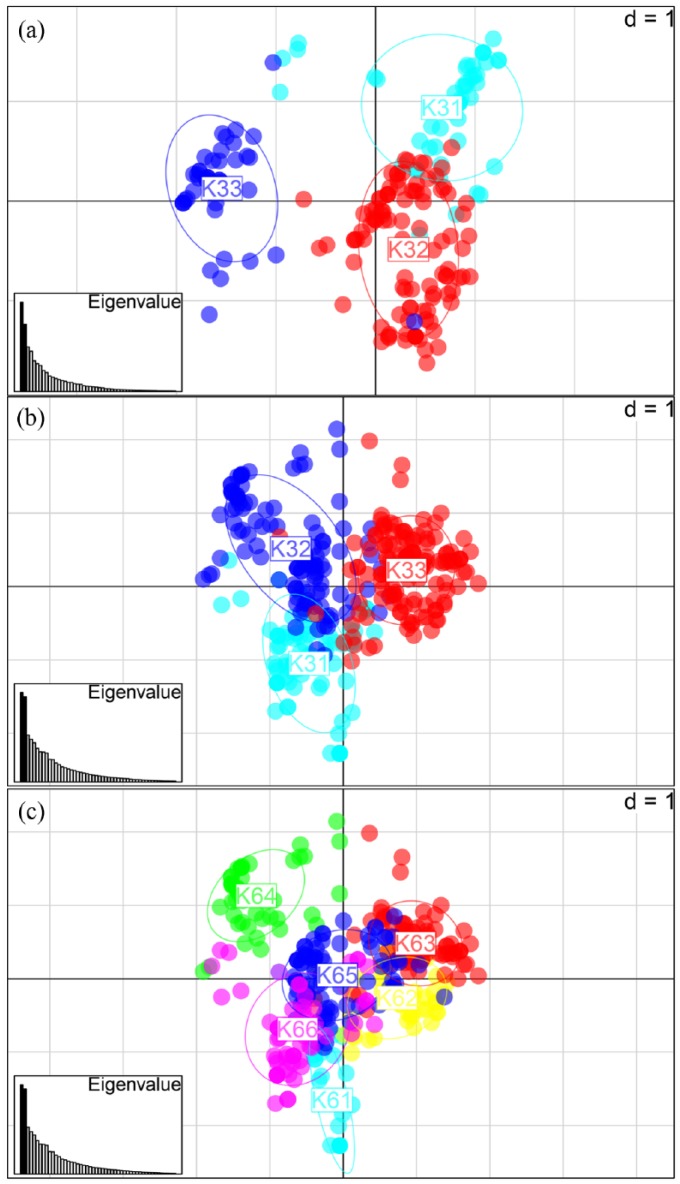
Principal components analysis output with samples coloured according to the *K* genepools from Instruct output (sample majority allocation). The axes represent the first two principal components in each case: (a) 195 Chinese samples, coloured according to *K* = 3 (see below), (b) 341 panEurasian samples, coloured according to *K* = 3 and (c) 341 panEurasian samples, coloured according to *K* = 6.

The first two principal components accounted for 16.3% and 12.3%, and 12.6% and 12.0%, for the Chinese and panEurasian datasets, respectively. Scatterplots of the first two principal components are shown in Figure 3a (Chinese dataset) and Figure 3b and c (panEurasian dataset). Samples are coloured according to their assignments to *K* populations under the Instruct clustering analysis. Clusters identified in Instruct (see below) show clear separation on the scatterplots in all cases.

### Bayesian clustering of microsatellite data

To explore signals of population substructure, we analysed the microsatellite data in two batches, first using only Chinese samples and second as a panEurasian dataset.

Analysis of the Chinese samples using Instruct showed a plateau for the value of ln*P*(*D*)_Chn_ from *K* = 7. The parameter Δ*K*_Chn_ reached a maximum at *K* = 3 and showed a smaller peak at *K* = 7. Correlations between replicate runs showed that estimates of *Q* were highly stable at *K* = 3, and we therefore present results from this model as capturing most of the structure in the data. For panEurasian samples, ln*P*(*D*)_Eur_ showed no clear plateau. Δ*K*_Eur_ showed a major peak at *K* = 3 and a second minor peak at *K* = 6. Both these values of *K* gave highly stable results among replicate runs, and we therefore present output for both models. The DIC reported the maximum value of *K* used in the analysis (*K* = 12) as optimal; this statistic has been little tested in Bayesian clustering analysis of genetic data, and in the light of the recommendation of Pritchard et al. (2010) to be conservative when selecting the optimal value of *K*, we discounted this parameter in favour of better tested methods.

**Figure 4.**
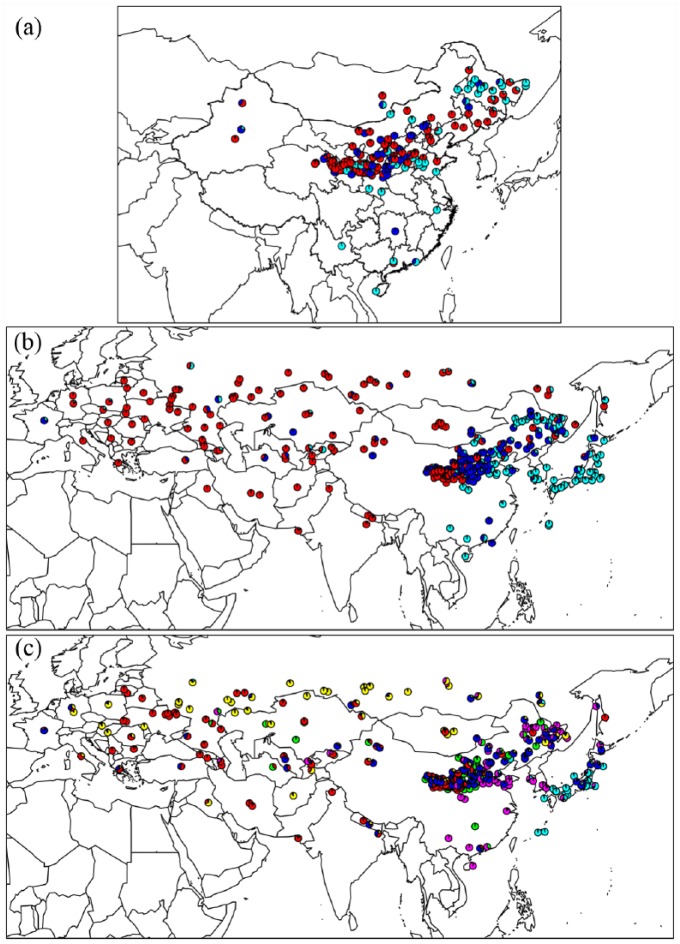
Proportional assignments of each landrace sample to ancestral genepools inferred using Instruct (Gao et al., 2007). Each sample is represented as a pie chart, mapped according to its origin as provided by the accession data supplied by the germplasm banks. Different colours of the pie slices represent the *K* genepools modelled by Instruct. Colours of the genepools are chosen to correspond with previously published analyses of related datasets (Hunt et al., 2011, 2013). The pie charts show the relative membership of the *K* genepools for each sample. The most realistic inferred values of *K* are shown: (a) 195 Chinese samples, for *K* = 3, (b) 341 panEurasian samples, under *K* = 3 and (c) 341 panEurasian samples, under *K* = 6.

Of the three clusters resolved in the Chinese dataset, one (cyan) shows a clear east, northeast and south Chinese distribution. Clusters 2 and 3 (red and blue, respectively) overlap in the Yellow River and Loess Plateau regions, with cluster 2 (red) showing a more western (upper Yellow River) focus (Figure 4a). This pattern within China is largely congruent with the three-cluster model from the larger pan Eurasian dataset. Beyond China, samples to the west and north, in Mongolia, Siberia, Central and South Asia, and Europe, have a strong predominant affinity with the red cluster, while samples in Korea and Japan belong to the cyan cluster (Figure 4b). We note that around one-third of the Chinese samples show altered cluster assignment between the Chinese and pan Eurasian dataset analyses; this is typical for Bayesian clustering algorithms, in which the identified groups depend on the information available from the particular sample set. When the Eurasian model is expanded to six clusters, we observe a north–south differentiation of central Asian/European populations, and there is some evidence of geographic differentiation within the middle and lower Yellow River region (Figure 4c).

### Modelling the origins of population expansions

Using our spatially explicit discriminative modelling approach, the most negative correlation values between distance from hypothesised origin location and genetic diversity for the China-only dataset are in northwestern China, approximately in the southeastern part of Gansu province (Figure 1a, negative correlation coefficients are shown in red and positive coefficients in blue). The sites of Dadiwan and Xinglonggou – two hypothesised source locations for the expansion of broomcorn millet, based on archaeobotanical evidence – are marked ‘1’ and ‘2’, respectively, on Figure 1a; correlations are more negative for Dadiwan. To test if the difference in correlation values between Dadiwan and Xinglonggou is more extreme than that expected by chance, we performed the permutation procedure described above (see ‘Methods’ section). This returned a two-tailed *p* value of 0.192 (see Figure 2a), suggesting that the data are insufficient to discriminate between these hypothesised source locations using this approach.

For the panEurasian dataset, the most negative correlation values between distance from hypothesised origin location and genetic diversity were for northeast Eurasia, and the most positive were for western Eurasia (see Figure 1b). Thus, under a model of a monotonic decline in genetic diversity with distance from origin location, our analyses do not support a western Eurasian origin for the expansion of broomcorn millet, but do admit the possibility of an eastern Eurasian origin. Two hypothesised source locations – the sites of Sokol’tsy and Xinglonggou – are indicated with marked ‘1’ and ‘2’, respectively. Again, we tested if the difference in correlations between these two sites is more extreme than that expected by chance (Figure 2b). We obtained a two-tailed *p* value of 0.108, indicating the data favour an eastern Eurasian origin for the expansion of broomcorn millet under the assumption of a monotonic decline in genetic diversity with distance from origin location, but that the difference in these values for these two sites only approaches significance.

### GBSSI genotyping

**Figure 5.**
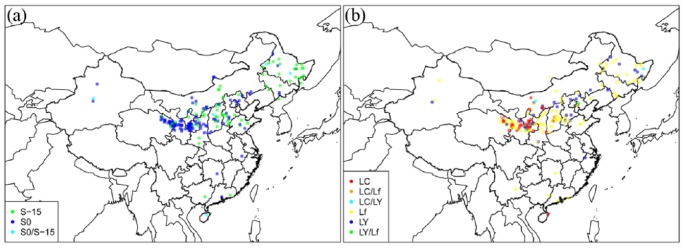
Geographical distribution of *GBSSI* genotypes for 195 Chinese landrace samples. (a) *GBSSI-S* locus. Samples shown as green points are homozygous wild type, that is, both alleles in the individual are the non-waxy S0. Samples shown as dark blue points are homozygous waxy, that is, both alleles are the mutant S-15. Samples shown as cyan points are heterozygous, that is, both alleles have one wild type (S0) and one waxy (S-15). The S0 allele is dominant, so heterozygous individuals are phenotypically wild type. (b) *GBSSI-L* locus. Samples shown as red points are homozygous for the wild-type (LC) allele. Samples shown as dark blue and yellow points are homozygous for different waxy mutations (LY and Lf, respectively). The three heterozygous combinations (LC/LY, LC/Lf and LY/Lf) are shown as cyan, orange and green points, respectively.

At the *GBSSI-S* locus, which is the major determinant of endosperm starch amylose and amylopectin composition, the wild-type S_0_ allele predominates to the south and west of the Yellow River, that is, Shaanxi, Ningxia, Gansu, Qinghai and Xinjiang provinces. The waxy mutant S_-15_ allele is at high frequency in the lower part of the Yellow River valley and northeast China (Figure 5a). At the *GBSSI-L* locus, for which waxy mutant alleles combine with S_-15_ to produce a fully waxy phenotype, the wild-type L_C_ allele has an upper Yellow River valley/western Loess Plateau distribution in western Shanxi, western Inner Mongolia, Ningxia, Gansu and Qinghai provinces. L_c_ does not occur east of Shanxi province. The waxy mutant alleles L_Y_ and L_f_ co-occur with L_c_ in this region but are both distributed throughout the sampled distribution in China, with L_f_ at higher overall frequency (Figure 5b).

## Discussion

### Evidence for an eastern Eurasian centre of origin of broomcorn millet

The question of whether cultivated broomcorn millet populations originated in China and/or central-eastern Europe (Jones, 2004) has stimulated much novel work in archaeobotany, genetics and stable isotope analysis across Eurasia. In our previous survey of microsatellite diversity in Eurasian *P. miliaceum* (Hunt et al., 2011), we suggested that the observed patterns of variation are somewhat more consistent with a Chinese origin and centre of dispersal, but we were unable to formally test this. As highlighted by Gerbault et al. (2014), many different evolutionary histories may give rise to a given genetic dataset with equal plausibility (equifinality); to discriminate between these histories, data should be tested for fit to a statistical model. Here, we use a simple model based on the assumption of a monotonic decline in genetic diversity with increasing distance from origin location (Manica et al., 2007; Ramachandran et al., 2005).

These analyses are supportive at a broad cross-continental scale of a Chinese centre of origin and dispersal of broomcorn millet. This result resonates with other proxies, which have also shifted the focus of early *P. miliaceum* exploitation firmly to the East, through both the accrual of positive evidence at early dates in China (Ren et al., 2016, and references therein) and comparative negative evidence further west (Lightfoot et al., 2013; Motuzaite-Matuzeviciute et al., 2013). Speculation about European, Caucasus or Central Asia origins for broomcorn millet arose from multiple sites in central and eastern Europe, and the Caucasus, apparently of comparable antiquity (pre-5000 BC) to those in China (reviewed in Hunt et al., 2008), and the lack of archaeobotanical research in Central Asia. In recent years, systematic flotation and direct dating of *Panicum* grains at sites from Neolithic cultures across northern China have vastly increased the evidence base for pre-5000 BC broomcorn millet with domesticated-type morphology here (Crawford et al., 2016; Gansu Provincial Institute of Cultural Relics and Archaeology (GPICRA), 2006; Lu et al., 2009; Tao et al., 2011; Wu et al., 2014; Yang et al., 2012; Zhang et al., 2012; Zhao, 2011, 2014; summarised in Ren et al., 2016). In contrast, direct dating of macrofossils from central and eastern Europe showed that their previous early Neolithic attributions were incorrect, and they date rather to ~1500 BC (the European Bronze Age) at the earliest (Motuzaite-Matuzeviciute et al., 2013). In the Caucasus, de novo excavations and re-evaluation of earlier reports have resulted in a similarly revised chronology for broomcorn millet, with the earliest firm evidence of the crop at 1200–1000 BC (Trifonov et al., 2017). Systematic archaeobotanical analysis in Central Asia has recovered *P. miliaceum* from sites dating from ~2200 BC (the Central Asian Bronze Age; Spengler et al., 2014).

This novel archaeobotanical work across Eurasia, implemented by several international research teams, has raised significantly the burden of proof for scientifically credible records of broomcorn millet, leading to an altered and much clearer picture of the crop’s chronology. Of the archaeobotanical record as it appeared in 2008, the main set of records still demanding re-evaluation (in terms of both identification and dating) is that of impressions in pottery from the territories to the north and west of the Black Sea, which are also ascribed to the early Neolithic (6400–5800 BC; Kotova, 2003).

New palaeodietary studies have complemented archaeobotanical advances by providing direct evidence for the role of millet in human and animal diets. A review of the palaeodietary literature from across Eurasia (Lightfoot et al., 2013) found evidence for isotopically detectable consumption of millet by some individuals in southern Europe in the 2nd millennium BC, with a stronger, population-level signal in central Europe during the 1st millennium BC.

In summary, the genetic evidence presented here, along with the evidence from archaeobotany and palaeodietary analysis, is now most consistent with a single origin of cultivated *P. miliaceum* somewhere in northern China, by at least the 6th millennium BC. This China-centric model resolves and supersedes the previous debate (Hunt et al., 2008; Jones, 2004) on the origins of broomcorn millet, in the absence of new evidence to the contrary.

### Evidence supporting a centre of origin of broomcorn millet in the western Loess Plateau of China

Within northern China, early (pre-5000 BC) archaeobotanical records of *P. miliaceum* come from several regional Neolithic cultures located in the ‘Chinese Fertile Arc’ (CFA; Ren et al., 2016. Primary data from Crawford et al., 2016; GPICRA, 2006; Lu et al., 2009; Office for Preservation, Municipality of Shenyang (OPMS) and Shenyang Palace Museum (SPM), 1985; Tao et al., 2011; Wu et al., 2014; Yang et al., 2012; Zhang et al., 2012; Zhao, 2011, 2014). We explored whether we could resolve a centre of origin of broomcorn populations from among these. The observed trend (Figure 2a) of higher negative correlation values between distance from hypothesised origin location and genetic diversity to the west of the range, although not statistically significant, is nonetheless suggestive and intriguing when taken in conjunction with the *GBSSI* genotype data (Figure 5b). The ancestral *GBSSI*-L_c_ allele is restricted to this region, while the mutant LY and Lf variants occur both here and in eastern and northeastern China. In principle, this distribution could result from either demographic history or strong selection against *GBSSI*-L_c_ in the east. However, the *GBSSI*-L genotype has only a modest effect on phenotype, which makes strong selection on this locus less likely (Hunt et al., 2013). An independent study on SSR variation in Chinese *P. miliaceum* accessions also found the Loess Plateau to be the region within China with the highest diversity (Hu et al., 2009). Although none of these data are conclusive, it all coheres with a centre of expansion of *P. miliaceum* somewhere in the western Loess Plateau.

### Possible relationships between population expansions and domestication

Our model assumes that *P. miliaceum*, including the immediate wild ancestor of broomcorn millet, underwent (at least one) range expansion at some point in the last 10,000–20,000 years. Although the past range of wild *P. miliaceum* is not known, we consider this is an uncontroversial assumption given the general global picture of shifting ranges both of vegetation types, for example, grasses, and of individual species, in the Holocene and terminal Pleistocene. It is the chronological relationship of this expansion (a plant population process) to cultivation (a human behavioural activity) or domestication (a human-driven evolutionary process) that is unknown. We can contrast two possible scenarios: first, a range expansion of wild *P. miliaceum* populations, followed by increasing human exploitation, cultivation and selection pressure resulting in the fixation of domestication traits in parallel in multiple populations around the CFA. In the southwest Asian Fertile Crescent, range expansions of wild cereals have been associated with the first part of the Younger Dryas period (Moore and Hillman, 1992). While we cannot pinpoint any single climatic event linked to expansion of wild millets in northern China, work on climate and vegetation dynamics shows considerable fluctuations of climate and vegetation types in this region in the last 20,000 years (Ni et al., 2014).

A second scenario is that the selection of domestication traits in a geographically localised region of the western Loess Plateau was followed by human-mediated dispersal across Neolithic north China. From the current dates, which place domesticated millet at Dadiwan as late or later than the easternmost sites in the CFA (Crawford et al., 2016; GPICRA, 2006; Lu et al., 2009; Ren et al., 2016; Tao et al., 2011; Wu et al., 2014; Yang et al., 2012; Zhang et al., 2012; Zhao, 2011, 2014), the first scenario appears the more plausible. This would imply that wild *P. miliaceum* had expanded across northern China before domestication by the mid-Holocene.

The wild origins of broomcorn millet remain a subject for speculation. Cytogenetic and phylogenetic analyses indicate that *P. capillare*, or a closely related species, was one of the diploid ancestors of the (wild) allotetraploid *P. miliaceum* (Hunt et al., 2014), presumably restricted to the Old World; however, given the understood New World native distribution (Tutin, 1980) of *P. capillare*, this finding only adds to the biogeographical mystery. The timing and location of the polyploidisation event that gave rise to *P. miliaceum* are unknown, as is the evolution of traits adapting the species to temperate semi-arid environments, from a predominantly tropical genus (Aliscioni et al., 2003). The unusually low genetic diversity of *P. miliaceum* could reflect a relatively recent, that is, Pleistocene, origin for the polyploid genome. At the broad scale, palynological reconstructions indicate that the vegetation of the Chinese Loess Plateau has alternated between glacial, C3-dominated steppe vegetation, and interglacial, C4-dominated humid grasslands in the last 150 ka (Vidic and Montañez, 2004), with the expansion of C4 plants driven by increasing summer temperatures and precipitation following the last glacial maximum (22–19 ka BP), from around 17 ka BP (Liu et al., 2005; Zhang et al., 2003). We can speculate that an expansion of wild-type *P. miliaceum*, laying down some of the modern-day genetic patterns, took place in this time frame.

Madsen and Elston (2007) postulated that early cultivation and selection pressure on wild millet occurred at the margins of its range, for example, at the northwest margin of the Loess Plateau, in the 8th millennium BC to provide a secure resource base in seasonally variable climates. This hypothesis is supported by the presence of lithic assemblages from the 10th millennium BC that include plant processing equipment. They implicitly assume that the distribution of wild millet was centred on and most productive in the Yellow River valley, echoing the Yellow River narrative for the origins of millet agriculture (Liu et al., 2009). The genetic data and analyses presented here indicate that the centre of range expansion, at some period, may in fact have been somewhat further to the west. The patterns of climate and vegetation change across north China in the Late Pleistocene and Holocene are highly complex, with substantial regional and local variation (Zhao et al., 2009); these results highlight the need for a more precise understanding of the ecophysiology of wild broomcorn millet that would enable modelling of its past distribution. Grassland ecosystems of China fall into four major types (Kang et al., 2007). According to Wang (2003), *Panicum ruderale* (= *P. miliaceum* subsp. *ruderale*) is only found in the most mesic (southeasterly) of these, the meadow-steppe. During the early Holocene, at least some parts of the northwest Loess Plateau and eastern Tibetan Plateau had a more humid climate and more mesic vegetation than today (Zhao et al., 2009), suggesting that elements such as wild millet could have flourished there. However, this was not the case at Dadiwan, where the early Holocene was drier and supported a desert-steppe vegetation (Zhao et al., 2009).

Although the details of climatic and vegetational change in northwestern China require further scrutiny, Madsen and Elston’s (2007) hypothesis for millet domestication resonates with the better-understood domestication of large-grained cereals in southwest Asia. There, the severe and abrupt climatic reversal of the Younger Dryas provoked rapid vegetational change on the timescale of a few centuries, with particular impact on the availability of diverse wild resources in semi-arid regions (Moore and Hillman, 1992). One strategy for mitigating the altered resource profile was management of wild grasses, imposing selection pressures that eventually resulted in the fixation of domestication traits.

Morphological traits that distinguish wild from domesticated *P. miliaceum* are poorly understood. The reported widespread panEurasian distribution of wild (or weedy) type *P. miliaceum* (defined by a shattering spikelet habit; Zohary et al., 2012) is not well substantiated in the floristic literature or herbarium collections (e.g. He et al., 2015). The archaeobotanical record of rachis fragments of millet is much inferior to those of larger grained cereals. Carbonised grains from, for example, phase 1 at Dadiwan (5800–5300 BC) and Xinglonggou (6000–5500 BC) have been inferred as domesticated on the basis of grain size and shape (Barton et al., 2009; Zhao, 2004) relative to wild *Panicum* spp. (Deng et al., 2015), but further study of *P. miliaceum* subsp. *ruderale*-type forms from China is needed. Morphological evidence from other cereals indicates that the transition to fully domesticated forms (fixation of non-shattering rachis alleles) took at least 2 to 3 millennia (Fuller et al., 2009); the archaeobotanical data for broomcorn millet are currently inadequate to infer where on the trajectory to domestication, the finds from widely dispersed sites in early 6th millennium BC might lie.

### A model for the panEurasian expansion of broomcorn millet

Returning to the pan-continental picture, if we accept that cultivated broomcorn millet in central Asia, central-western Russia and Europe originated in China, the microsatellite and *GBSSI* data strongly imply that the ultimate source of these populations was in western China, around the southwest Loess Plateau. The northwestward expansion of broomcorn cultivation, evidenced by the archaeobotanical record, is thus inferred to have a relatively local origin, contrary to the assumption of, for example, Zhou et al. (2016) that it came from ‘the middle and lower reaches of the Yellow River’. Broomcorn millet is found at numerous sites up to 2500 m altitude in the northeastern Tibetan Plateau (NETP), adjacent to the Loess Plateau, from ~3200 BC, belonging to the late Yangshao, Majiayao, Qijia, Xindian and Kayue cultures (Chen et al., 2015). From 2500 BC, it is one of the principal crops in the Hexi corridor to the north of the NETP (Zhou et al., 2016). These authors speculate that cooling climate drove millet down to lower altitudes and then eventually restricted cultivation in the Hexi corridor. Broomcorn appears in the Bronze Age in Xinjiang at Xiaohe (~1500 BC; Yang et al., 2014), and from Begash in eastern Kazakhstan at 2200 BC (Frachetti et al., 2010; Spengler et al., 2014), with accumulating evidence from central Asia then indicating it followed an ‘Inner Asian Mountain Corridor’ route towards the Caspian basin (Miller et al., 2016) and across modern-day Turkey.

Patterns of genetic variation across central Asia and Siberia indicate some north–south differentiation (Figure 4c), and we can speculate that the northernmost populations represent a secondary phase of westward expansion, which could date from the late second to first millennia BC. There is a lack of macrofossil data for northern Kazakhstan and southern Siberia, but carbon isotope evidence of enriched d13C values at sites in the Altai–Tuva–Khakassia regions suggests millet cultivation by *c.* 1400 BC and particularly in the early first millennium BC (Murphy et al., 2013; Svyatko et al., 2013). Isotopic data from Chalcolithic/Bronze Age northern Kazakhstan (2900–1400 BC) are negative for a millet signal (Matuzeviciute et al., 2015; Ventresca Miller et al., 2014); no Iron Age data are yet available.

Despite these new data for central Asia and eastern Siberia, the task still remains of joining up the millet routeways to Europe. Valamoti (2016) reports large concentrations of broomcorn millet from late 3rd millennium BC Skala Sotiros in northern Greece, but the grain has not been directly dated. This is imperative, as its apparent presence here predating the 2nd millennium route charted by Miller et al. (2016) is puzzling, though material from some of the Near Eastern sites also requires chronological confirmation.

Although our focus in this article has been the dispersal of cultivated broomcorn millet to central Asia and Europe, its expansion to other regions of Asia also deserves attention. On the Far Eastern rim, archaeobotanical evidence indicates that *P. miliaceum* reached both the Russian Far East (Primor’ye) by *c.* 3500 BC in the Zaisanovka culture (Kuzmin, 2013) and the Korean peninsula at a similar time in the Middle Chulmun period (Crawford and Lee, 2003; Lee, 2011). Broomcorn millet in Japan dates to the Final Jomon period (mid-1st millennium BC) in southern Honshu (Nasu and Momohara, 2016). Its appearance in Japan is approximately contemporaneous with rice, suggesting both cereals were introduced as a package from Korea (Nasu and Momohara, 2016). The genetic clusters that dominate Korea and Japan (shown in pink and light blue in Figure 4c) are closely related (Hunt et al., 2011, 2013), supporting this route of dispersal. However, a number of landraces from Hokkaido and northern Honshu are genetically similar (dark blue in Figure 4c) to those from the far northeast of China (Heilongjiang). Broomcorn millet, along with several other crops, appears in the Okhotsk culture on Hokkaido in the mid-1st millennium AD. The genetic patterns support the idea that broomcorn millet, like barley, had a second independent introduction to Japan from the Russian Far East (Crawford, 2011; Leipe et al., 2017).

On a continental scale, our analyses have clarified the Holocene biogeography of *P. miliaceum*. The species’ greatest genetic diversity is in the western Loess Plateau of China, the region of origin from where all the world’s *P. miliaceum* ultimately derived. Homing in on North China and the fixation of domestication traits, the pattern could be clarified by a clearer understanding of those traits and how to recognise them, in the context of different models of how that fixation may have proceeded. However, our analyses offer no support for a separate west Eurasian location as a centre of population expansion.

## Supplemental Material

Table_S1_Chinese_Panicum_samples_and_genotypes_050218 – Supplemental material for Genetic evidence for a western Chinese origin of broomcorn millet (Panicum miliaceum) Click here for additional data file. Supplemental material, Table_S1_Chinese_Panicum_samples_and_genotypes_050218 for Genetic evidence for a western Chinese origin of broomcorn millet (Panicum miliaceum) by Harriet V Hunt, Anna Rudzinski, Hongen Jiang, Ruiyun Wang, Mark G Thomas and Martin K Jones in The Holocene
